# FaDA: A web application for regular laboratory data analyses

**DOI:** 10.1371/journal.pone.0261083

**Published:** 2021-12-20

**Authors:** Richard Danger, Quentin Moiteaux, Yodit Feseha, Estelle Geffard, Gérard Ramstein, Sophie Brouard

**Affiliations:** 1 Nantes Université, CHU Nantes, Inserm, Centre de Recherche en Transplantation et Immunologie (CRTI), UMR 1064, ITUN, Nantes, France; 2 Université de Nantes, LS2N DUKe, UMR6004, Centrale Nantes, IMT Atlantique, INRIA and CNRS, Nantes, France; 3 LabEx IGO “Immunotherapy, Graft, Oncology”, Nantes, France; University of Nebraska-Lincoln, UNITED STATES

## Abstract

Web-based data analysis and visualization tools are mostly designed for specific purposes, such as the analysis of data from whole transcriptome RNA sequencing or single-cell RNA sequencing. However, generic tools designed for the analysis of common laboratory data for noncomputational scientists are also needed. The importance of such web-based tools is emphasized by the continuing increases in the sample capacity of conventional laboratory tools such as quantitative PCR, flow cytometry or ELISA instruments. We present a web-based application FaDA, developed with the R Shiny package that provides users with the ability to perform statistical group comparisons, including parametric and nonparametric tests, with multiple testing corrections suitable for most standard wet-laboratory analyses. FaDA provides data visualizations such as heatmaps, principal component analysis (PCA) plots, correlograms and receiver operating curves (ROCs). Calculations are performed through the R language. The FaDA application provides a free and intuitive interface that allows biologists without bioinformatic skill to easily and quickly perform common laboratory data analyses. The application is freely accessible at https://shiny-bird.univ-nantes.fr/app/Fada.

## Introduction

Increasing numbers of web-based data analysis and visualization tools have been developed using the R programming package Shiny [1] and made available to researchers. Despites other programming languages are suitable for web-based applications, these tools rely on the well-recognized usefulness of R to analyze data from different perspectives, especially for statistical analysis, and provide interactive visualizations. Hence, Shiny tools are enabling wet-laboratory researchers the ability to take advantage of bioinformatics advancements [2].

While they are free and save the user time in the analytic stages without requiring that the user have extensive computational skills, most of the current online Shiny applications are dedicated to specific objectives or technologies, such as *shinyheatmap* to generate heatmaps for large datasets [3], *shinyCircos* to build Circos plots from genomic data [4], *iDEP* for RNAseq data analysis [5] or *shinyGEO* to analyze gene expression datasets directly from the Gene Expression Omnibus (GEO) repository [6]. Applications for data generated from common laboratory techniques such as quantitative PCR, flow cytometry or enzyme-linked immunosorbent assay (ELISA) are also needed. Technological advances in these methods have allowed researchers to generate significant data output. Flow cytometry technologies can run a large number of samples with a tenth of fluorochrome parameter combinations. In addition, multiplex ELISAs can produce readings for up to ten cytokines per well, and advances in quantitative PCR (qPCR) devices have allowed the analysis of samples in less than an hour. These high-volume data outputs leave laboratory researchers with a time-consuming data analysis process. First analysis steps often start with description of the dataset, group comparisons between groups of interest and analysis of correlated parameters. Heatmap, principal component analysis (PCA) graph and correlogram allow to visualise the entire datasets and to identify potential subsets or outliers. In parallel, individual graphics, either in dots, whiskers or violins allow to explore individual parameters. For such analyses, researchers usually perform targeted parameter analysis with several hands-on processes, increasing the risks of information loss and human error.

In order to give the possibility to explore research datasets, rapidly, with an easy-to-use tool, we created a free, user-friendly and interactive Shiny web application supporting regular laboratory analyses of a wide array of data, including flow cytometry and qPCR data. This multi-tool utility suite in R Shiny allows researchers to perform classical statistical group comparisons, including parametric and nonparametric tests with multiple testing correction and to produce heatmaps, PCA, receiver operating curves (ROCs) and correlogram visualizations. The FaDA application is freely accessible at https://shiny-bird.univ-nantes.fr/app/Fada

## Methods

### FaDA application

**Fig 1:**
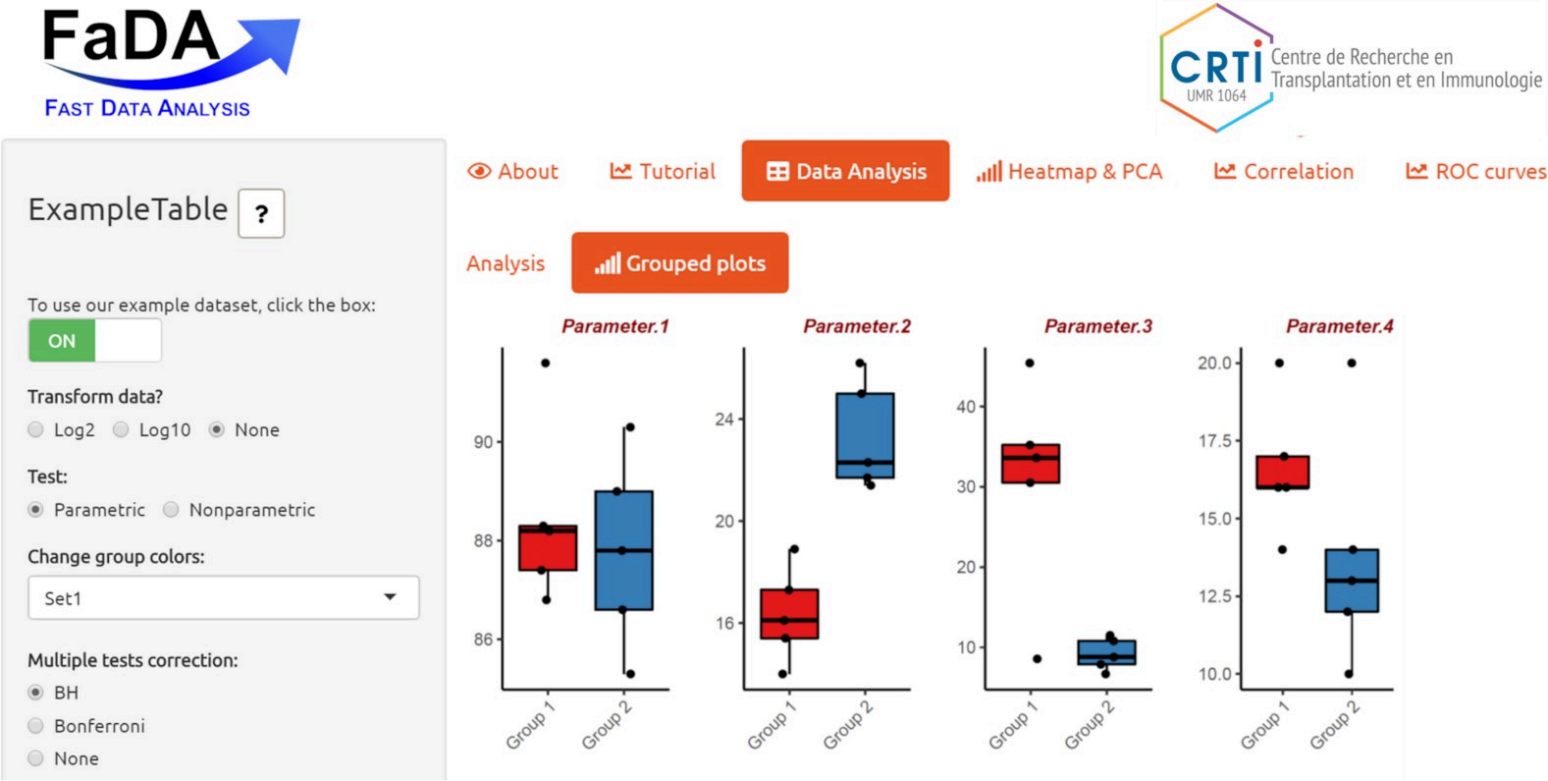
Overview of the FaDA application display. Upon data upload, users are automatically directed to the *Data Analysis* tabset (highlighted in orange) to view the statistical summary of their dataset. The 6 different tabsets are available in the main panel (*About*, *Tutorial*, *Data Analysis*, *Heatmap & PCA*, *Correlation*, and *ROC Curves*) while the sidebar displays various options related to data transformation (log10 or log2), statistical analysis (parametric or nonparametric, multiple correction options) and data visualisation options (group colors, graph options) depending on the selected tabset. In this figure, the “Grouped plots” subtab is selected, displaying whisker plots of the preloaded example dataset.

FaDA was developed in R (release 3.6.1, http://www.rproject.org) [7] and implemented as a web application using the R Shiny package (version 1.4.0) from R Studio (http://shiny.rstudio.com). As an open-source application, the FaDA code is available through GitHub at https://github.com/danger-r/FaDAapp. FaDA was dockerized using Docker software (https://www.docker.com/) and made available through ShinyProxy on a Linux server (CentOS 7 with 12 Go RAM allowed for FaDA) hosted at the BiRD bioinformatics core facility within the University of Nantes (https://pf-bird.univ-nantes.fr/). FaDA uses integrated work frames of R packages allowing an intuitive interface. A complete list of the packages used may be found in S1 Table. The interface layout is built using the *shiny* and *shinythemes* packages with a sidebar for user interaction and six main panels (*About*, *Tutorial*, *Data Analysis*, *Heatmap & PCA*, *Correlation* and *ROC curves*), with subtabs available within these panels (Fig 1).

### Data upload and file input

The application starts with the *About* page, which displays the general and background information of the application. The sidebar provides a simple demonstration dataset, including virtual data from 2 groups with 5 samples each, to explore the features of the web application. Alongside, user can upload and analyse its own dataset, appropriately formatted. While FaDA can process files with thousands of values, analysis of such large datasets should be performed using a local version to improve interactivity thanks to the source code available through GitHub. Data are uploaded in a text format (tabular-delimited ‘.txt’ or ‘.csv’ file), with a point or a coma as a decimal separator, with a file limit size of 5 Mo. To allow for flexible use of the application with minimal preparation time, sample identification is in columns or rows. FaDA input only requires unique names for sample identification, and the second row or column is named “Group” to identify sample group labels.

Users can find the *Tutorial* page explaining how to prepare dataset. Furthermore, the *Tutorial* page displays explanations of the tools with statistical tests available through FaDA with recommendations.

### Statistical summary

FaDA initially formulates a descriptive statistical summary after the upload of a dataset. The statistical analysis table presents mean and standard deviation or median and interquartile interval (IQR) per group as parametric or nonparametric options, respectively. The p-value of the Shapiro-Wilk normality test indicates whether the distribution of the dataset differs from a Gaussian distribution guiding the users toward parametric or nonparametric tests. The data can be log2 or log10 transformed, which is notably useful for gene expression datasets. Group comparisons are performed using parametric t.tests or ANOVA with Tukey’s ‘honest significant difference’ method for multiple group comparisons. Welch’s t.test is proposed in case of unequal variance. Alternatively, the Mann-Whitney test or the Kruskal-Wallis test with Dunn’s test of multiple comparisons using the *FSA* package are available as nonparametric group comparisons [8]. To correct for false positives due to multiple testing, p-value statistical corrections are performed with the Bonferroni or Benjamini & Hochberg (BH) methods [9]. For more readability, a sliding bar allows user to highlight significative values below the selected threshold.

### Graph visualization plots

Shiny allows for built-in support of interactive graph plots of data using R’s graph representative and graph plot packages *gplots* and *ggplots2* [10]. The available graph plots include box-and-whiskers, points, individual bars, grouped bars and violins plots and users can define the Y axis origin to 0 instead of the automatic level. Using the *plotly* package [11], interactive features are displayed, including zooming, panning, selecting, and downloading plots as png image files. Heatmap data representation is available as either static or interactive. A static heatmap, may be customized by adding sample hierarchical clustering and color schemes using the *ComplexHeatmap* package [12]. PCA allows the display of a covariance matrix and PCA plots to identify potential outliers or sample clustering. In cases of missing values, imputation is performed using the ten nearest neighbor averaging with the *impute* package [13]. Both heatmaps and PCA plots are visualized in an interactive mode using the *heatmaply* and *plotly* packages, respectively [11,14].

### Correlation analysis

To assess the correlations among parameters, the correlation coefficients are summarized in a correlogram thanks to the *corrplot* package [15]. Individual correlation graphs display scatter plots of two selected parameters with the correlation of these two parameters given. Correlation coefficients (r) and statistical significance tests are calculated either with the parametric Pearson correlation or the Spearman nonparametric methods. Since complete observations are used to calculate the correlations, the ten nearest neighbour averaging method is used to impute missing values [13]. Association (r) or significative values can be highlighted in bold and yellow for more readability using the sliding bars in the sidebar.

### ROC curves

Receiver operating characteristics (ROC) curves, area under the curve (AUC) and associated parameters can be viewed on the ROC curve tab using the *pROC* package [16]. Several ROC curves can be added on the same plot for comparison.

## Results

Two examples are provided to exhibit various possibilities offered by FaDA and evidencing that results from FaDA are consistent with previous analyses, with gene expression and flow cytometry data, two major methods used in biology research. Data can be formatted in row or column which is compatible with different measurement outputs; only the addition of a row or column indicating group identification is needed before upload, reducing preparation time for users. The 2 case studies also evidenced the interactive creation of heatmap, PCA graph and correlogram with customizable options including colorization and clustering.

### Case study 1 –gene expression data

**Fig 2:**
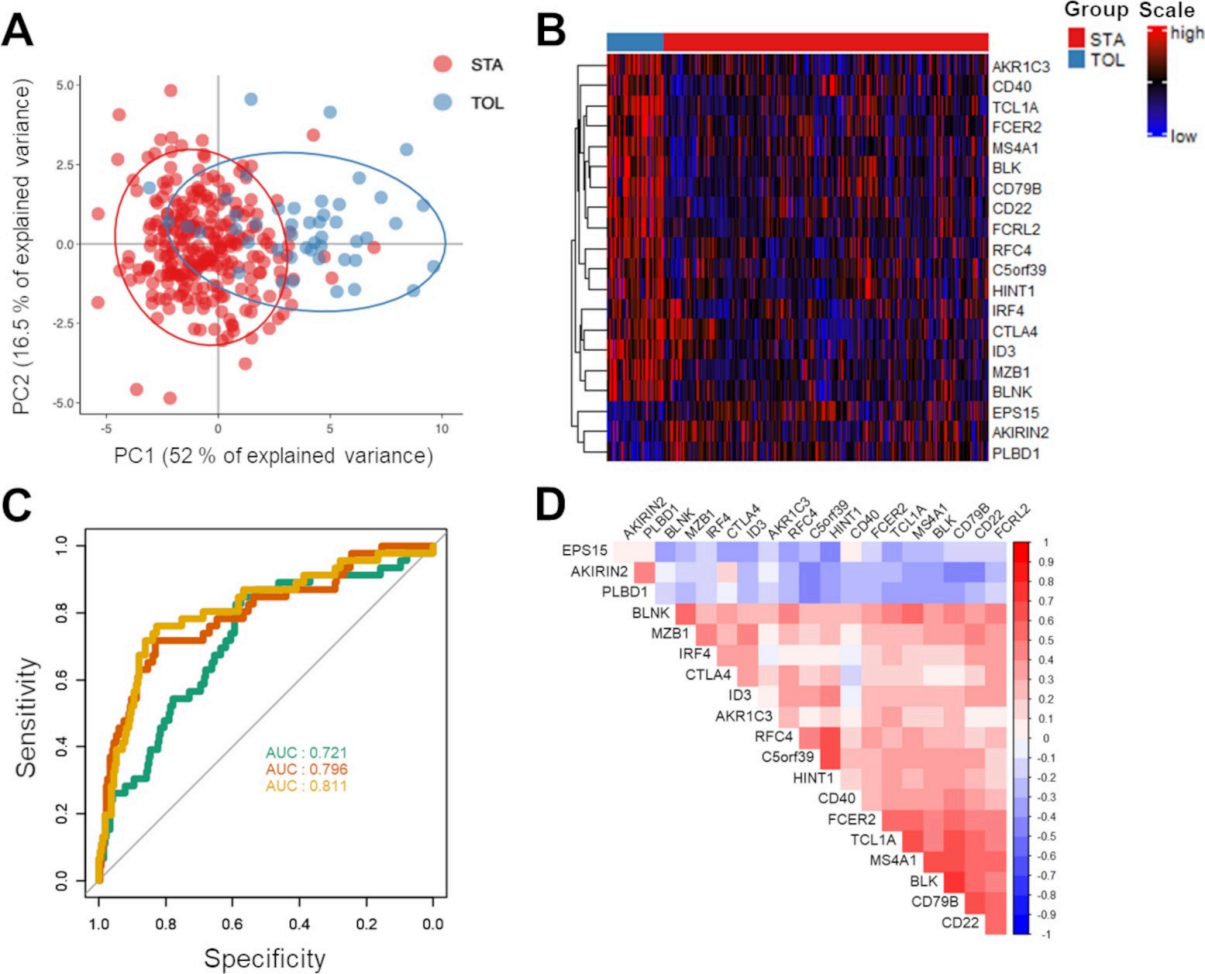
Data analysis of a 20-gene expression dataset from renal transplant patients. A) PCA and B) heatmap plots highlighting the clear difference in gene expression between the two groups, TOL (blue) and STA (red). C) ROC curves of groups displaying the AUCs of the selected genes, *AKR1C3*, *AKIRIN2* and *CD22*. D) Gene-gene correlation analysis using a correlogram to highlight groups of genes.

We used a 20-gene expression dataset from peripheral blood from two groups of renal transplant patients: 46 operational tolerant patients who stopped their immunosuppressive regimen while maintaining a stable renal function and 266 renal transplant patients with stable function under immunosuppression [17]. This matrix was already normalized (mean-centered log-intensity values divided by the standard deviation), so no transformation, *e*.*g*. log2 transformation, was applied. Given the gene expression matrix, FaDA allows clear discrimination of the two populations of patients using heatmap and PCA visualization (Fig 2A and 2B). The first component of the PCA (PC1) explained 52% of the observed variance. ROC curves analysis highlighted individual genes able to discriminate both populations with AUCs above 0.7, such as the *AKR1C3* gene, which reached an AUC of 0.796 (Fig 2C). The correlogram allows the identification of correlated genes *MS4A1*, *CD22*, *CD79B*, *FCRL2*, *BLK* and *TCL1A* (Fig 2D), in accordance with the previous signature found for operational tolerance and the implication of B cells [18]. In addition, FaDA provides same values for means of STA and TOL, raw p-values of standard t.tests and AUCs from ROC curves comparing STA and TOL than commercial GraphPad Prism (v. 9.1.0) or Microsoft Excel softwares with less time-consuming data manipulation (S2 Table).

### Case study 2 –flow cytometry data

**Fig 3:**
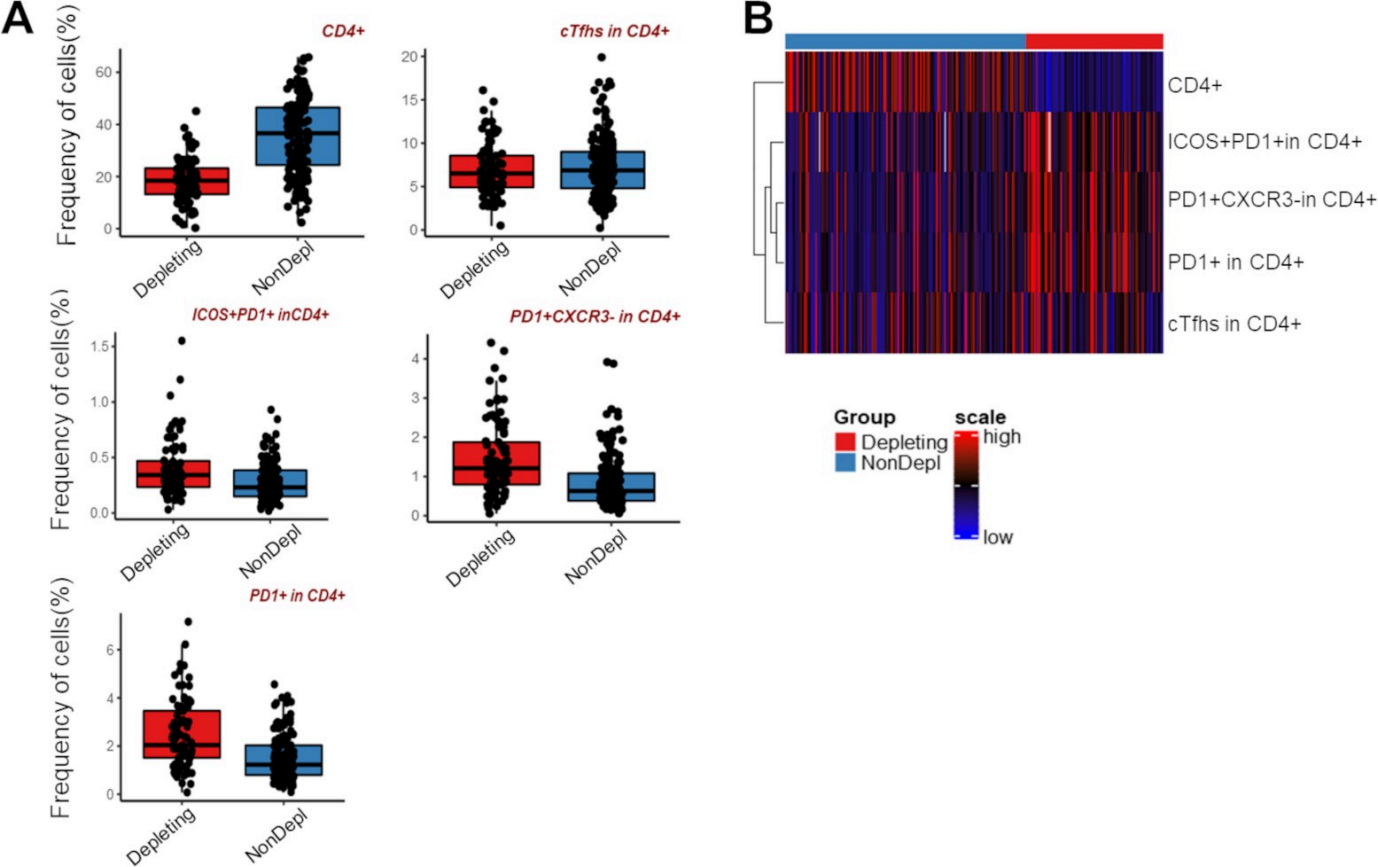
Cytometric dataset analysis using FaDA. A) Whisker plots of patients receiving ATG-depleting induction treatment (Depleting; n = 87) and basiliximab nondepleting treatment (n = 145) or the absence of induction therapy (n = 5) (NonDepl) on the frequency of CD4^+^ cells, total cTfh^+^ and cTfh subsets, namely CXCR5^+^PD1^+^, CXCR5^+^PD1^+^ICOS^+^ and CXCR5^+^PD1^+^CXCR3^-^, at one-year post-transplantation. B) Heatmap graph presenting a visual summary of the flow cytometry data.

We benefited from a previous study that aimed to characterize circulating follicular T helper cells (cTfhs) in the peripheral blood of renal transplant patients [19]. We reported a notable impact of anti-thymocyte globulin (ATG)-depleting induction treatment (n = 87) compared to basiliximab nondepleting treatment (n = 145) or the absence of induction therapy (n = 5) on the frequency of total CD4^+^ lymphocytes and on the activated cTfh subsets, namely CXCR5^+^PD1^+^, CXCR5^+^PD1^+^ICOS^+^ and CXCR5^+^PD1^+^CXCR3^-^, at one year after transplantation. Using FaDA, we can exhibit here, consistent with what was previously shown, that patients with depleting treatment exhibited lower levels of total CD4^+^ lymphocytes but higher frequencies of activated cTfh subsets using Benjamini-Hochberg multiple test correction (adjusted p-value <0.0001, Fig 3A, S3 Table). The heatmap of the dataset exhibits higher levels of activated cTfh subsets in the depletion treatment group (Fig 3B).

## Discussion

Here, we demonstrate FaDA as an easy-to-use and helpful application for the analysis of commonly generated data from flow cytometry and gene expression microarrays. The FaDA web application is free and user-friendly, provided for scientists lacking computational skills to easily and rapidly perform data analysis; while reducing the error arising from the hands-on data analysis regularly performed by wet-laboratory researchers. The FaDA application allows users to benefit from various data visualization options to intuitively understand the results of the data analysis, identifying significant findings and possibly highlighting outliers with limited time consumption. Interestingly, while we designed FaDA for biologic data, analyses and visualisations provided can be used in others domains requiring similar analyses. We used two case studies from previously published datasets [17,19] to demonstrate the usefulness of FaDA for analysing data commonly generated by biological and medical researchers, such as microarray and flow cytometry datasets, two major methods used in biology research. Nevertheless, as designed for a general purpose and for noncomputational biologist scientists, advanced analyses such as time series or time-dependant analyses, will require other tools. The R Shiny library has been specifically built to implement web-based applications from R language, a powerful tool for data analysis and especially statistical analyses [7]. However, interactivity is limited with R and other programming languages, such as Python or Perl, would also have been well-suited for creating interactive visualizations. In particular, complete customization of figures would require other tools than FaDA which is not its primary utility. Despites, previous tools have been successfully implemented in R Shiny for large datasets and complex analyses, such as for RNAseq or scRNAseq analyses [3,5,20,21], the purpose of FaDA is not to perform heavy-lifting bioinformatic analysis that would results in substantial time to analysis. In such cases, advanced users will either use a local version thanks to the source code available through GitHub or dedicated software along with their bioinformatic and statistical skills. Although FaDA supports various statistical options and help text is provided, either in the *Tutorial* page or as mouseover texts, it cannot replace recommendations from statisticians that users may need for particular cases, as for any type of statistical analysis software. As an open-source application, code is available for any user, notably with R expertise. We are offering user support for FaDA and we also plan to continue to develop this application providing additional tests and visualisation tools.

## Supporting information

S1 Table List of R/Shiny packages. (DOCX)

S2 Table From case study 1, FaDA provides same values than commercial GraphPad Prism (v. 9.1.0) or Microsoft Excel software with less time-consuming data manipulation. (DOCX)

S3 Table From the second case study, FaDA provides same values than commercial GraphPad Prism (v. 9.1.0) or Microsoft Excel software with less time-consuming data manipulation. (DOCX)

## Abbreviations

AUC: Area Under the Curve
FaDA: Fast Data Analysis
GEO: Gene Expression Omnibus
ELISA: enzyme-linked immunosorbent assay
PCA: Principal Component Analysis
qPCR: quantitative PCR
ROC: Receiver Operating Curve
