# Supplementary material for: FaDA: A web application for regular laboratory data analyses
Source: PLoS One. 2021 Dec 20;16(12):e0261083. doi: 10.1371/journal.pone.0261083 (PMC8687579; doi:10.1371/journal.pone.0261083)
Supplement: S1 Table — (DOCX) [file pone.0261083.s001.docx]

**Supplementary Table S1:** List of R/Shiny Packages

| **Package Name** | **Use** | **Version** | **Reference** | **Link/Reference** |
| --- | --- | --- | --- | --- |
| **shiny** | Web Application Framework for R | 1.4.0 | Winston Chang, Joe Cheng, JJ Allaire, Yihui Xie and Jonathan McPherson (2019). shiny: Web Application Framework for R | https://CRAN.R-project.org/package=shiny |
| **shinythemes** | Themes for Shiny | 1.1.2 | Winston Chang (2018). shinythemes: Themes for Shiny | https://CRAN.R-project.org/package=shinythemes |
| **shinycssloaders** | Load spinner | 0.2.0 | Andras Sali (2017). shinycssloaders: Add CSS Loading Animations to 'shiny' Outputs | https://CRAN.R-project.org/package=shinycssloaders |
| **shinyBS** | Tooltips, popovers and alerts | 0.61 | Eric Bailey (2015). shinyBS: Twitter Bootstrap Components for Shiny | https://CRAN.R-project.org/package=shinyBS |
| **shinyWidgets** | custom widgets | 0.5.0 | Victor Perrier, Fanny Meyer and David Granjon (2019). shinyWidgets: Custom Inputs Widgets for Shiny. | https://CRAN.R-project.org/package=shinyWidgets |
| **data.table** | Table import (fread function) | 1.14.0 | https://rdatatable.gitlab.io/data.table/ | https://CRAN.R-project.org/package=data.table |
| **ggplot2** | Plot graphs | 3.2.1 | H. Wickham. ggplot2: Elegant Graphics for Data Analysis. Springer-Verlag New York, 2016. | https://cran.r-project.org/web/packages/ggplot2/index.html |
| **plotly** | Plot interactives graphs | 4.9.1 | Carson Sievert (2018) plotly for R. | https://plotly-r.com |
| **gridExtra** | arrange multiple grid-based plots | 2.3 | Baptiste Auguie (2017). gridExtra: Miscellaneous Functions for "Grid" Graphics | https://CRAN.R-project.org/package=gridExtra |
| **grid** | Grid display | 3.6.1 | Murrell, P. (2005) R Graphics. Chapman & Hall/CRC Press. | https://github.com/pmur002/gridgraphics |
| **gplots** | For color panels in the heatmap | 3.0.1.1 | Gregory R. Warnes, Ben Bolker, Lodewijk Bonebakker, Robert Gentleman, Wolfgang Huber, Andy Liaw, Thomas Lumley, Martin Maechler, Arni Magnusson, Steffen Moeller, Marc Schwartz and Bill Venables (2019). gplots: Various R Programming Tools for Plotting Data. | https://CRAN.R-project.org/package=gplots |
| **ComplexHeatmap** | Creating heatmaps | 2.2.0 | Gu, Z. Complex heatmaps reveal patterns and correlations in multidimensional genomic data. Bioinformatics 2016 | https://github.com/jokergoo/ComplexHeatmap |
| **circlize** | Color visualisation with *ComplexHeatmap* | 0.4.8 | Gu, Z. circlize implements and enhances circular visualization in R. Bioinformatics 2014 | https://cran.r-project.org/web/packages/circlize/index.html |
| **heatmaply** | For interactive heatmaps | 1.0.0 | Tal Galili, Alan O'Callaghan, Jonathan Sidi, Carson Sievert; heatmaply: an R package for creating interactive cluster heatmaps for online publishing, Bioinformatics, 2017 | https://cran.r-project.org/web/packages/heatmaply/index.html |
| **corrplot** | Creating correlograms | 0.84 | Taiyun Wei and Viliam Simko (2017). R package "corrplot": Visualization of a Correlation  Matrix | https://github.com/taiyun/corrplot |
| **impute** | knn imputation on the PCA when NA values are presents | 1.60.0 | Trevor Hastie, Robert Tibshirani, Balasubramanian Narasimhan and Gilbert Chu (2019). impute: impute: Imputation for microarray data | http://www.bioconductor.org/packages/release/bioc/html/impute.html |
| **FSA** | Used for Dunn test | v0.8.26 | Ogle, D.H., P. Wheeler, and A. Dinno. 2019. FSA: Fisheries Stock Analysis | https://github.com/droglenc/FSA |
| **DT** | To create datatables | 0.10 | Yihui Xie, Joe Cheng and Xianying Tan (2019). DT: A Wrapper of the JavaScript Library 'DataTables' | https://CRAN.R-project.org/package=DT |
| **RColorBrewer** | Generating color palettes | 1.1-2 | Erich Neuwirth (2014). RColorBrewer: ColorBrewer Palettes | https://CRAN.R-project.org/package=RColorBrewer |
| **pROC** | To create and analysis ROC curves | 1.15.3 | Xavier Robin, Natacha Turck, Alexandre Hainard, Natalia Tiberti, Frédérique Lisacek,  Jean-Charles Sanchez and Markus Müller (2011). pROC: an open-source package for R and S+ to analyze and compare ROC curves. BMC Bioinformatics | https://cran.r-project.org/web/packages/pROC/index.html |
