# Supplementary material for: FaDA: A web application for regular laboratory data analyses
Source: PLoS One. 2021 Dec 20;16(12):e0261083. doi: 10.1371/journal.pone.0261083 (PMC8687579; doi:10.1371/journal.pone.0261083)
Supplement: S2 Table — (DOCX) [file pone.0261083.s002.docx]

**Supplementary Table S2**: From case study 1, FaDA provides same values than commercial GraphPad Prism (v. 9.1.0) or Microsoft Excel software with less time-consuming data manipulation.

|  |  | *AKIRIN2* | *AKR1C3* | *BLK* | *BLNK* | *C5orf39* | *CD22* | *CD40* | *CD79B* | *CTLA4* | *EPS15* |
| --- | --- | --- | --- | --- | --- | --- | --- | --- | --- | --- | --- |
| **GraphPad Prism** | TOL mean | -0.791 | 1.174 | 1.177 | 1.477 | 0.813 | 1.380 | 0.766 | 1.357 | 1.031 | -1.059 |
|  | STA mean | -0.00661 | -0.00463 | -0.00336 | 0.00152 | -0.00606 | 0.01035 | 0.00207 | 0.00457 | -0.00860 | -0.00158 |
|  | raw p value | 0.000002 | <0,000001 | <0,000001 | <0,000001 | <0,000001 | <0,000001 | 0.000002 | <0,000001 | <0,000001 | <0,000001 |
|  | ROC AUC | 0.722 | 0.796 | 0.774 | 0.789 | 0.734 | 0.811 | 0.730 | 0.799 | 0.760 | 0.764 |
|  |  |  |  |  |  |  |  |  |  |  |  |
| **FaDA** | TOL mean | -0.791 | 1.174 | 1.177 | 1.477 | 0.813 | 1.380 | 0.766 | 1.357 | 1.031 | -1.059 |
|  | STA mean | -0.00661 | -0.00463 | -0.00336 | 0.00152 | -0.00606 | 0.01035 | 0.00207 | 0.00457 | -0.00860 | -0.00158 |
|  | raw p value | 1.52E-06 | 9.19E-13 | 7.66E-12 | 5.26E-15 | 2.48E-07 | 1.39E-14 | 1.89E-06 | 2.12E-14 | 3.91E-10 | 1.52E-10 |
|  | ROC AUC | 0.722 | 0.796 | 0.774 | 0.789 | 0.734 | 0.811 | 0.730 | 0.799 | 0.760 | 0.764 |
|  |  |  |  |  |  |  |  |  |  |  |  |
| **Microsoft Excel** | TOL mean | -0.791 | 1.174 | 1.177 | 1.477 | 0.813 | 1.380 | 0.766 | 1.357 | 1.031 | -1.059 |
|  | STA mean | -0.00660 | -0.00463 | -0.00336 | 0.00152 | -0.00606 | 0.01035 | 0.00207 | 0.00457 | -0.00860 | -0.00158 |
|  | raw p value | 1.520E-06 | 9.190E-13 | 7.664E-12 | 5.256E-15 | 2.481E-07 | 1.389E-14 | 1.887E-06 | 2.120E-14 | 3.910E-10 | 1.521E-10 |
|  | ROC AUC | na | na | na | na | na | na | na | na | na | na |

|  |  | *FCER2* | *FCRL2* | *HINT1* | *ID3* | *IRF4* | *MS4A1* | *MZB1* | *PLBD1* | *RFC4* | *TCL1A* |
| --- | --- | --- | --- | --- | --- | --- | --- | --- | --- | --- | --- |
| **GraphPad Prism** | TOL mean | 1.063 | 1.346 | 0.958 | 1.516 | 0.721 | 1.124 | 1.555 | -0.985 | 1.111 | 1.716 |
|  | STA mean | 0.00843 | 0.00852 | -0.00098 | 0.00195 | 0.01032 | 0.00134 | 0.00283 | -0.01111 | -0.00827 | 0.00414 |
|  | raw p value | <0,000001 | <0,000001 | <0,000001 | <0,000001 | 0.000006 | <0,000001 | <0,000001 | <0,000001 | <0,000001 | <0,000001 |
|  | ROC AUC | 0.744 | 0.823 | 0.756 | 0.729 | 0.826 | 0.813 | 0.828 | 0.735 | 0.806 | 0.876 |
|  |  |  |  |  |  |  |  |  |  |  |  |
| **FaDA** | TOL mean | 1.063 | 1.346 | 0.958 | 1.516 | 0.721 | 1.124 | 1.555 | -0.985 | 1.111 | 1.716 |
|  | STA mean | 0.00843 | 0.00852 | -0.00098 | 0.00195 | 0.01032 | 0.00134 | 0.00283 | -0.01111 | -0.00827 | 0.00414 |
|  | raw p value | 8.03E-10 | 2.42E-14 | 2.20E-08 | 8.79E-17 | 6.16E-06 | 6.70E-12 | 3.34E-17 | 1.25E-08 | 5.34E-11 | 6.30E-23 |
|  | ROC AUC | 0.744 | 0.823 | 0.756 | 0.826 | 0.729 | 0.813 | 0.828 | 0.735 | 0.806 | 0.876 |
|  |  |  |  |  |  |  |  |  |  |  |  |
| **Microsoft Excel** | TOL mean | 1.063 | 1.346 | 0.958 | 1.516 | 0.721 | 1.124 | 1.555 | -0.984 | 1.111 | 1.716 |
|  | STA mean | 0.00843 | 0.00851 | -0.00098 | 0.00195 | 0.01032 | 0.00133 | 0.00283 | -0.0111 | -0.00827 | 0.00414 |
|  | raw p value | 8.029E-10 | 2.424E-14 | 2.204E-08 | 8.786E-17 | 6.163E-06 | 6.697E-12 | 3.339E-17 | 1.252E-08 | 5.339E-11 | 6.288E-23 |
|  | ROC AUC | na | na | na | na | na | na | na | na | na | na |

For each of the 20 genes are displayed: means of STA and TOL groups, raw p-values of standard t.tests assuming equal variance and AUCs from ROC curves comparing STA and TOL.
