# Supplementary material for: FaDA: A web application for regular laboratory data analyses
Source: PLoS One. 2021 Dec 20;16(12):e0261083. doi: 10.1371/journal.pone.0261083 (PMC8687579; doi:10.1371/journal.pone.0261083)
Supplement: S3 Table — (DOCX) [file pone.0261083.s003.docx]

**Supplementary Table S3**: From the second case study, FaDA provides same values than commercial GraphPad Prism (v. 9.1.0) or Microsoft Excel software with less time-consuming data manipulation.

|  |  | CD4+ | cTfhs in CD4+ | ICOS+PD1+ in CD4+ | PD1+ in CD4+ | PD1+CXCR3- in CD4+ |
| --- | --- | --- | --- | --- | --- | --- |
| **GraphPad Prism** | Depleting mean | 18.38 | 7.07 | 0.40 | 2.45 | 1.45 |
|  | NonDepl mean | 35.08 | 7.35 | 0.28 | 1.50 | 0.86 |
|  | raw p value | <0,0001 | 0.535 | <0,0001 | <0,0001 | <0,0001 |
|  |  |  |  |  |  |  |
| **FaDA** | Depleting mean | 18.38 | 7.07 | 0.40 | 2.45 | 1.45 |
|  | NonDepl mean | 35.08 | 7.35 | 0.279 | 1.50 | 0.860 |
|  | raw p value | 5.4E-19 | 0.535 | 1.9E-05 | 3.4E-09 | 1.2E-07 |
|  |  |  |  |  |  |  |
| **Microsoft Excel** | TOL mean | 18.38 | 7.066 | 0.402 | 2.450 | 1.446 |
|  | STA mean | 35.08 | 7.351 | 0.279 | 1.499 | 0.860 |
|  | raw p value | 5.4E-19 | 0.535 | 1.9E-05 | 3.4E-09 | 1.2E-07 |

For frequency of CD4^+^ cells, total cTfh^+^ and cTfh subsets, namely CXCR5^+^PD1^+^, CXCR5^+^PD1^+^ICOS^+^ and CXCR5^+^PD1^+^CXCR3^-^, at one-year post-transplantation, are displayed: means and raw p-values of standard t.tests assuming equal variance comparing patients receiving ATG-depleting induction treatment (Depleting; n=87) and basiliximab nondepleting treatment (n=145) or the absence of induction therapy (n=5) (NonDepl).
